# Physical Activity Influences Negative Emotion Among College Students in China: The Mediating and Moderating Role of Psychological Resilience

**DOI:** 10.3390/healthcare13101170

**Published:** 2025-05-17

**Authors:** Lijun Zuo, Yao Wang, Jinfu Wang, Yidi Yao, Guan Yang

**Affiliations:** 1 School of Physical Education, South China University of Technology, Guangzhou 510641, China; spljzuo@mail.scut.edu.cn (L.Z.); 202420141786@mail.scut.edu.cn (Y.W.); 202320142029@mail.scut.edu.cn (J.W.); 2 School of Design, South China University of Technology, Guangzhou 510006, China

**Keywords:** physical activity, negative emotion, psychological resilience, mediating effect, moderating effect, Chinese college students

## Abstract

**Background/objective**: Negative emotion, such as anxiety and depression, significantly impacts college students’ academic performance, social relationships, and overall well-being. Given that, this study aims to explore the effect of physical activity on alleviating negative emotion among Chinese college students and to examine the mediating and moderating mechanisms of psychological resilience including individual power and supportive power. **Methods**: A cross-sectional survey was conducted with 596 college students from 10 universities in Guangdong Province (M*_age_* = 20.32, SD = 1.47). Data were collected using the Physical Activity Rating Scale (PARS-3), Adolescent Psychological Resilience Scale (APRS), and Depression–Anxiety–Stress Scale (DASS-21). Descriptive and correlation analysis, hierarchical regression analysis, and PROCESS macro models were used to test the mediating and moderating effects. **Results**: (1) Physical activity (*β* = −0.312, *p* < 0.001) and supportive power (*β* = −0.391, *p* < 0.001) both significantly negatively predicted negative emotion; (2) Individual power played a partial mediating role between physical activity and negative emotion (indirect effect accounted for 31.4%, 95% CI [−0.395, −0.242]); (3) Supportive power moderated the relationship between physical activity and negative emotion (interaction effect *β* = 0.089, *p* < 0.01), with a stronger effect of physical exercise on reducing negative emotion under low supportive conditions (*b* = −0.401 vs. *b* = −0.224). **Conclusions**: Physical activity not only directly affects negative emotion but also indirectly influences negative emotion by enhancing psychological resilience (individual power), and supportive power could moderate this effect through a resource substitution mechanism. The present study provides a theoretical basis for designing targeted mental health interventions and emphasizes the synergistic effect of integrating physical activity and social support on college students’ mental health.

## 1. Introduction

The mental health of college students has emerged as a critical global public health concern, with extensive empirical evidence underscoring the pervasive detrimental effects of negative emotion, defined pervasively as certain transient psychological states (e.g., anxiety, depression, anger), which are distinct from chronic mental disorders. Epidemiological studies report alarming prevalence rates: approximately 17% of university students meet diagnostic criteria for major depressive disorder [1,2,3], while 29%, 27%, and 24% exhibit clinically significant levels of depression, anxiety, and stress, respectively, as measured by the Depression, Anxiety, and Stress Scale (DASS-21) [4,5]. These emotional challenges substantially impair academic performance, social functioning, and overall well-being [6,7,8], a phenomenon exacerbated by the intensely competitive academic environments and sociocultural pressures uniquely faced by Chinese students. Hence, identifying effective strategies to mitigate negative emotion represents an urgent priority.

As we all know, officially defined as any bodily movement requiring energy expenditure (e.g., running, swimming, and other resistance training), physical activity has gained recognition as a potent intervention means for individuals’ emotion regulation [8,9]. Meta-analytic evidence consistently demonstrates its therapeutic efficacy in alleviating symptoms of depression, anxiety, and stress [10,11], with moderate-intensity exercise exhibiting particularly robust effects in reducing emotional distress among college populations [12,13]. Building on this evidence mentioned above, we naturally proposed Hypothesis 1 (H1): College students’ engagement in regular physical activity may be inversely associated with levels of negative emotion.

The relationship between physical activity and emotional well-being, however, is not unidimensional. Psychological resilience—usually defined as the capacity to adaptively navigate adversity and maintain functional stability under stress [14]—may serve as a critical mediator or moderator. Specifically, grounded in the process model of resilience [15], this construct encompasses two dimensions: individual power (internal factors, such as goal-directed focus and emotional regulation) and supportive power (external resources including familial and interpersonal support). These dimensions align with Dodge et al.’s emotion regulation framework [16], which distinguishes between intrinsic (self-initiated) and extrinsic (environmentally scaffolded) regulatory mechanisms. Exercise psychology further posits that physical activity could effectively enhance personal cognitive flexibility and attention control [11], potentially fostering resilience and thereby attenuating vulnerability to negative emotion [17]. Accordingly, we pointed out Hypothesis 2 (H2): Individual power could mediate the relationship between physical activity and negative emotion among college students.

Notably, marked disparities in emotional outcomes persist even among college students with comparable exercise engagement, suggesting the influence of additional moderating factors. Even though prior research has predominantly examined resilience as a mediator, its role as a moderator and particularly the supportive power dimension, remained unknown until now. Preliminary evidence indicates a strong inverse association between resilience and negative emotion [18], consistent with the resource substitution hypothesis [17,18], which posits that individuals may compensate for deficient external support through health-promoting behaviors, such as physical activity. Therefore, we reasonably put forward Hypothesis 3 (H3): Supportive power might moderate the association between physical activity and negative emotion, with the protective effects of exercise being amplified under conditions of low external support.

**Figure 1:**
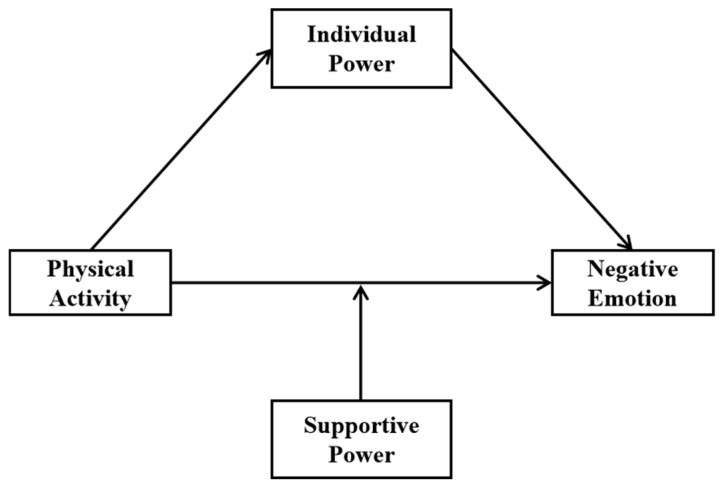
The assumed relationship model of four main variables in this study.

Given those mentioned above, the present study aims to investigate the dual mechanisms—mediation (via individual power) and moderation (via supportive power)—through which physical activity affects negative emotion among Chinese college students, and then, the following hypothesis has been proposed, namely physical activity might be negatively associated with negative emotion of college students, and individual power may play a mediating role between physical exercise and NE, as well as supportive power might moderate the relationship between them (seen in Figure 1). By integrating theoretical frameworks from exercise psychology and resilience theory, our findings aim to inform the design of targeted, multi-level interventions to enhance mental health outcomes in this population.

## 2. Materials and Methods

### 2.1. Participants and Procedure

This study adopted a cross-sectional survey design and followed the principle of random sampling, and selected 700 college students from 10 universities in Guangdong Province as the survey respondents to objectively evaluate their physical activity, negative emotion, and psychological resilience through several standardized scales. The sample size of the present work was determined by the 10-times rule, namely the number of questionnaire will be ten times that of all survey items [19,20], so 530 participants should be chosen by us. Meanwhile, given that there is a 30% dropout rate in practical surveys, the final recruitment target was adjusted to nearly 700 participants. The survey was conducted anonymously, without revealing or infringing on any privacy issues of the respondents, and the questionnaires were collected after completion in full accordance with the voluntary principle. At last, after careful and serious checking by us, 4 questionnaires were found to be problematic, 78 questionnaires had inconsistent or incomplete responses, and 22 questionnaires were directly excluded due to those subjects possibly having several mental health issues. Thus, the effective sample size of this study was 596 in final, with an overall recovery rate of 85.14%. The age range of the subjects was 18–22 years (20.32 ± 1.47 years), with 314 males (52.7%) and 282 females (47.3%).

The study was conducted in accordance with the 1964 Declaration of Helsinki and its later amendments or comparable ethical standards and, meanwhile, also approved by the Institutional Review Board of the South China University of Technology, involving minimal risk and anonymous survey procedures, and all subjects were asked to obtain written informed consent before participating in this investigation.

### 2.2. Measurement

#### 2.2.1. Physical Activity Rating Scale

Physical activity was evaluated using the Physical Activity Rating Scale (PARS-3) originally developed by Liang and Liu [21]. This scale contains three items: exercise intensity, exercise duration, and exercise frequency, each measured using a 5-point Likert scale (1–5). The total physical exercise score was calculated using the formula: Intensity × (Duration − 1) × Frequency, yielding a possible range of 0 to 100 points, with higher scores indicating greater physical activity level. The PARS-3 has already demonstrated good test-retest reliability (*r* = 0.82). In this study, the scale showed satisfactory internal consistency with a Cronbach’s *α* of 0.678.

#### 2.2.2. Depression-Anxiety-Stress Scale

Negative emotion was objectively assessed using the Chinese version of the Depression–Anxiety–Stress Scale (DASS-21), originally developed by Lovibond and Lovibond [22] and translated by Yuan [23]. The scale comprises three subscales: depression, anxiety, and stress, each containing 7 items. Responses were recorded on a 4-point-Likert scale (0-3), with total scores ranging from 0 to 63. Higher scores indicate more severe negative emotional states. In this study, the overall scale demonstrated excellent internal consistency (*α* = 0.956), and coefficient for each sub-scale being 0.884 (depression), 0.878 (anxiety), and 0.910 (stress), respectively.

#### 2.2.3. Psychological Resilience

Psychological resilience was measured by the Adolescent Psychological Resilience Scale (APRS) developed by Hu and Gan [15]. This 27-item scale contains five dimensions: the first three assess individual resilience factors (goal focus, emotional control, and positive cognition), while the latter two measure supportive factors (family support and interpersonal assistance). Items are rated on a 5-point Likert scale (1–5), with higher total scores indicating greater resilience. The scale demonstrated good internal consistency in this study (*α*= 0.841), with coefficient for each sub-scale being 0.751 for individual factors and 0.682 for supportive factors.

In addition, several essential demographic information, including age and sex, was also collected using a standard survey questionnaire.

### 2.3. Statistical Analysis

All statistical analyses were performed using IBM SPSS Statistics (version 26.0) with a predetermined significance threshold of *p* < 0.05, 0.01, and 0.001. Continuous variables were expressed as the mean ± standard deviation (M ± SD), and categorical variables were displayed as frequency (*n*) and percentage (%). The analytical procedure consisted of three sequential steps: Firstly, bivariate correlations between key study variables were examined using Pearson’s correlation analysis. Secondly, a mediation analysis was conducted through the PROCESS macro (Model 4) to determine whether psychological resilience’s personal strength component mediates the relationship between physical activity (independent variable) and negative emotion (dependent variable). Thirdly, a moderation analysis was implemented using PROCESS macro (Model 1) to evaluate whether psychological resilience’s supportive factors moderate the association between physical activity and negative emotion, and then 5000 bootstrap resamples was utilized to generate 95% bias-corrected confidence intervals (CI). A significant mediation or moderation effect was established when the upper-level and lower-level 95% CI of the effect value excluded zero [24]. For significant moderation effects, simple slope analysis was subsequently performed to probe interaction effects by calculating regression lines at higher (M + 1 SD) and lower (M − 1 SD) levels of the moderator. Prior to the moderation analyses, the key variables were all standardized to facilitate coefficient interpretation and also to decrease collinearity among main variables.

## 3. Results

### 3.1. Test for Common Method Variance

After data collection, Harman’s single-factor test was used to statistically verify the potential serious common method bias. The results showed that there were five common factors with eigenvalues greater than 1, and the variance explained by the first common factor was only 20.38%, which is significantly less than the critical value standard of 40% [25,26]. Therefore, this study does not have a serious common method bias problem.

### 3.2. Descriptive Statistics and Correlation Analysis

The results of descriptive statistics and correlation analysis are shown in Table 1. As is shown below, the average score of college students’ physical exercise amount was 16.08, which was located in the stage of the light-level exercise. Simultaneously, their self-reported score of negative emotion, IP and SP were all in the moderate-level range, along with scores of 51.52, 41.12, and 31.11, respectively.

In terms of correlation analysis, the Pearson’s correlation coefficients of the physical activity, negative emotion, IP, and SP were obviously represented. The PA was negatively correlated with NE (*r* = −0.394, *p* < 0.001) but positively associated with IP *(r* = 0.387, *p* < 0.001) and SP (*r* = 0.300, *p* < 0.001). Likewise, the NE was inversely related to SP (*r* = −0.464, *p* < 0.001) and IP (*r* = −0.423, *p* < 0.001). In addition, IP and SP were also positively associated with each other (*r* = 0.569, *p* < 0.001).

### 3.3. The Mediating Effect Analysis

Table 2 exhibits hierarchical regression analyses performed to identify the relative explanatory variance of three sets with demographic indicators, PA, and IP to predict the degree of negative emotion.

Table 2 shows the mediating effect of IP between PA and negative emotion via stepwise regression analysis. In step 1, in the regression analysis of IP on PA, PA was a positive predictor of NE (*β* = 0.387, *t* = 10.225, *p* < 0.001), and the model was significant overall (*F* = 104.545, *p* < 0.001). At the same time, the 95% CI [0.313, 0.461] excluded 0, and PA explained 15% of the variance in IP. In step 2, together they explained 24.1% of the variance in negative emotion, along with both PA and IP, which could negatively predict negative emotion (*β* = −0271, *p* < 0.001; *β* = −0.319, *p* < 0.001).

**Figure 2:**
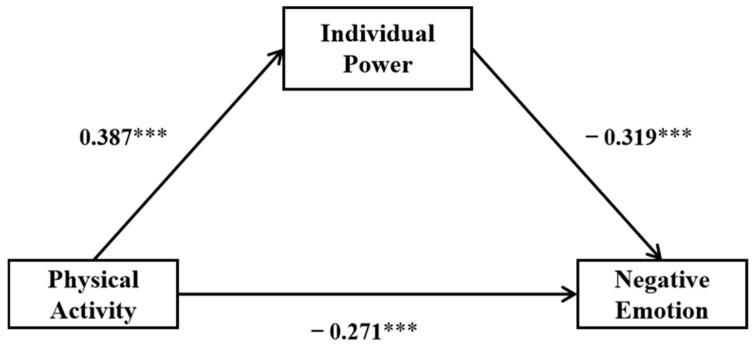
The mediation path of physical activity on negative emotion by individual power. *** *p* < 0.001.

As is clearly shown in Figure 2, IP played a partial mediating role in the relationship between PA and negative emotion, and the indirect effect was −0.124, which means PA could decrease negative emotion by improving IP. The results revealed that IP was really a mediator between PA and negative emotion and partially controlled the course by which PA decreased negative emotion.

### 3.4. The Moderating Effect Analysis

To determine whether the relation between PA and negative emotion was moderated by SP, the PROCESS macro Model 1 was carried out to certify the moderating effect underlying this correlation. The main results are presented in Table 3.

As can be seen from analyses of the moderating effect, PA was a significant and negative predictor of NE (*β* = −0.312, *t* = −8.205, *p* < 0.001). Similarly, SP was also a negative predictor of NE (*β* = −0391, *t* = −10.758, *p* < 0.001), which means increasing PA and SP could decrease NE. Finally, the interaction effect of PA and SP had a significant effect for NE (*β* = 0.089, *t* = 2.752, *p* < 0.01). The confidence interval for PA was [−0.387, −0.237], which does not encompass the value of zero, further substantiating its statistical significance. Similarly, the confidence interval for SP was [−0.463, −0.319] (exclude 0), confirming its significance.

In summary, the table demonstrates that both PA and SP exerted a significant positive influence on the reduction of NE. Although the magnitude of the interaction effect between PA and SP may be modest, it was statistically significant. These findings will be of considerable importance for understanding how the management of NE can be enhanced through the augmentation of PA and the bolstering of SP.

**Figure 3:**
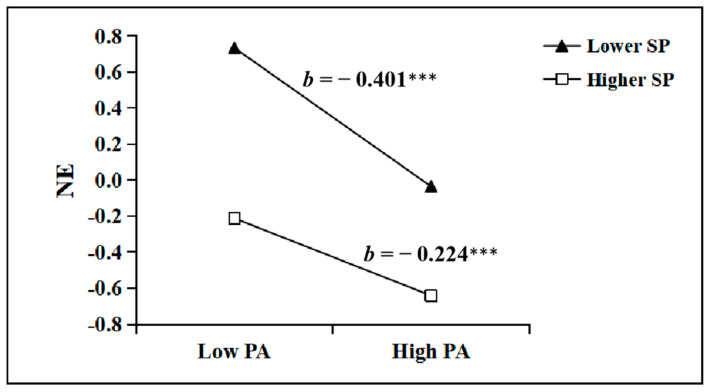
The relationship between PA (physical activity) and NE (negative emotion) at lower and higher levels of SP (supportive power). *** *p* < 0.001.

Figure 3 illustrated the relationship between PE and NE, taking into account the moderating effect of the level of SP. Simple slope analysis indicated that physical activity was negatively associated with NE under both high SP (*b* = −0.224) and low SP (*b* = −0.401) conditions, and these associations were significant (*p* < 0.001). This suggests that under conditions of lower SP, increasing PA can effectively mitigate NE. In contrast, under conditions of higher SP, the inverse relationship between PA and NE was attenuated. These results may indicate that engaging in PA would be greatly beneficial for reducing NE among college students. SP serves a moderating role between PA and NE, indicating that the impact of PA on NE may vary depending on the level of SP.

## 4. Discussion

The primary objective of the current study was to investigate the mediating and moderating mechanism that underlies the relationship between PA and NE in college students. Our findings revealed that PA could not only directly affect NE but also indirectly influences them by bolstering individual psychological resilience. Specifically speaking, the IP of psychological resilience emerged as a pivotal mediator in this relationship, and furthermore, the SP of psychological resilience was identified as a significant negative predictor of NE, indicating that increased levels of both PA and SP could independently attenuate negative emotional states. Meanwhile, the interaction between PA and SP was found to significantly affect NE. The results of this study have perfectly demonstrated our hypotheses and also provided compelling evidence to elucidate the nexus among PA, NE, and psychological resilience, including both dimensions of individual power and supportive power.

Previous research has underscored an escalating prevalence of psychological disorders among college students, including depression and anxiety, which are indicative of negative emotional states [27,28,29]. This issue has become a critical focus within the realms of psychological and behavioral science, garnering heightened scholarly attention. A constellation of NE is known to precipitate a decline in academic performance, diminished sleep quality, and life satisfaction among college students, potentially culminating in physical maladies or even mortality [27,30]. Consequently, effectively mitigating NE among college students presents a substantial and pressing challenge that demands resolution. Prior studies have indicated that engaging in moderate to vigorous physical activity at least three times per week elicits more pronounced intervention effects on depression, anxiety, and stress responses [31,32,33,34]. Consistent with Hypothesis 1, PA exhibited a direct negative association with NE, aligning with meta-analytic evidence that regular exercise reduces depressive and anxious symptoms [35,36].

More critically, IP emerged as a significant mediator, accounting for 31.4% of the total effect. This supports the stress-buffering model [37], which posits that resilience resources attenuate emotional distress by enhancing adaptive coping. Specifically, PA may foster IP through mechanisms, such as improved cognitive flexibility [38] and attentional disengagement from ruminative thoughts, thereby equipping students to navigate academic and social stressors, thus confirming Hypothesis 2. Furthermore, this study has identified that the variable of psychological resilience (SP) moderates the impact of PA on NE among college students. It is important to note that, as illustrated in Figure 2, under conditions of lower SP, the ameliorative effect of PA on NE is more pronounced. This suggests that in the absence of adequate support, the enhancement of physical activity yields a more significant improvement in emotional well-being. This pivotal finding may imply that, around the context of insufficient external support, individuals may increasingly rely on PA to elevate their mood and psychological health. Consequently, encouraging and promoting PA could be an effective strategy to assist individuals in low-support environments in improving their emotional state. However, this does not diminish the significance of PA under conditions of higher SP.

While both PA and SP independently reduced NE, their interaction revealed a compensatory dynamic: PA exerted stronger effects under low SP (Figure 2). This aligns with the resource substitution hypothesis [39], which suggests that individuals in low-support environments may rely more heavily on personal health behaviors (e.g., exercise) to compensate for deficient external resources. Conversely, high SP likely provides alternative coping avenues (e.g., social connectedness), diluting the necessity of PA for emotional regulation. According to the dynamic model of psychological resilience [40], external protective factors from family, school, and peer groups are also potential forces that promote individual psychological resilience. Based on the supportive factors of psychological resilience, the higher the level of support from parents and friends, the stronger the psychological resilience of college students, and the lower the baseline level of NE. Therefore, the effect of PA on the NE of college students may be weakened. This indicates that both SP and PA play the crucial role in emotional regulation, potentially complementing each other to a varying degree and collectively fostering psychological health, thereby confirming Hypothesis 3.

Our mediation findings resonate with Chang’s work on childhood adversity [41], where resilience partially mediated the maltreatment–depression link. However, their focus on trauma contrasts with our emphasis on routine stressors (e.g., academic pressure), highlighting resilience’s universal applicability. Similarly, Poole et al. and Tian et al., identified resilience as a protective factor against depression [42,43], but our study promotes this by specifying how resilience operates—through both internal (IP) and external (SP) pathways—and how PA activates these pathways. Notably, according to the stress-buffering hypothesis [44], social support can alleviate people’s perception of environmental crises and anxiety, and a latest study conducted during the pandemic also corroborates this finding [45]. The modest yet significant interaction effect of PA and SP, challenges the assumption that social support universally amplifies health behaviors’ benefits. Instead, our results suggest a nuanced interplay where SP and PA serve complementary, context-dependent roles. This echoes recent calls to integrate multi-level resilience frameworks [46], and also acknowledges that emotional well-being arises from both individual agency and environmental burden.

The findings suggest practical steps for mental health initiatives at colleges and universities. For students in low-support environments, such as those lacking familial or peer networks, structured physical education programs could be particularly beneficial [47,48], as exercise might compensate for scarce external resources and support their mental health. Universities can further enhance this support by integrating mindfulness and cognitive-behavioral techniques into physical education, which aligns with evidence that combined physical-psychological interventions yield superior outcomes [7,49]. Additionally, fostering social support through all kinds of mentorship programs and community-building activities can complement the positive effects of exercise. While social capital enhances the emotional benefits of physical education, it does not replace them; thus, creating a supportive environment is crucial for improving students’ overall mental well-being in daily life.

Despite the valuable insights discussed above, it is essential to acknowledge certain limitations inherent in this study. Firstly, the cross-sectional design of this research allows us to identify associations between variables but does not enable us to establish causality, which implies that we cannot definitively determine whether PA leads to a reduction in NE or if individuals with lower levels of NE are more inclined to engage in PA. Given that, the longitudinal design or experimental intervention could be conducive to help us address this problem. Secondly, the study’s sample, consisting of university students from Guangdong Province, may limit the generalizability and external validity of the findings. University students from different regions, with diverse cultural backgrounds and educational levels, may exhibit varying patterns of physical activity and psychological resilience. Thirdly, although the PARS-3 demonstrated an acceptable reliability with an internal consistency coefficient of 0.678, the relatively low internal consistency might be attributed to the brevity of the scale, which includes only three items. Given that, other more effective means should be employed by us or other researchers to measure university students’ physical activity. Fourthly, the interaction effect between PA and SP is statistically significant but also modest; thus, more empirical studies should be performed to prove its reliability and validity in coming days.

Lastly, and also equally important, while this study has revealed the mediating role of psychological resilience between PA and NE, as well as the moderating effect of SP, the mechanisms and specific processes underlying these effects may require further elucidation through future experimental research. It would also be beneficial to consider incorporating other variables that may impact NE, such as sleep quality, dietary habits, social support, and personal life events in future studies. Especially an essential and potential variable, that is various types of physical activity, as a key moderator, it may significantly influence the relationship between physical activity and negative emotion. Conducting more additional cross-sectional and longitudinal research will greatly enhance the accuracy and reliability of the findings from this study. In addition, it must be also pointed out that a potential bidirectional relationship may exist between physical activity and negative emotion. Even though physical activity could directly affect negative emotion and indirectly by psychological resilience, in actuality, negative emotion may be also a key variable to influence physical activity. Given that, the future research should further examine the latent bidirectional association between physical activity and negative emotion by a cross-lagged design.

## 5. Conclusions

The present work aims to elucidate the underlying relationships among physical activity, psychological resilience—encompassing personal strength and social support—and negative emotion. A particular focus is directed towards investigating whether individual power acts as a mediator between physical activity and negative emotion and if supportive power exerts a moderating influence on this relationship. This work finally concludes with two significant findings. One is that individual power is posited to mediate the relationship between physical activity and negative emotion, and the other one is that supportive power is suggested to moderate the relationship between them. In essence, the study posits that the interplay among physical activity, individual power, and supportive power is a critical determinant of negative emotion among university students. The findings underscore the importance of integrating physical activity into students’ lives not only for its direct benefits but also for its potential to bolster personal strength and leverage social support, thereby fostering a more resilient emotional state. However, it is crucial to temper these conclusions with the acknowledgment that further research is warranted to substantiate these relationships. And future studies should employ diverse methodologies, including longitudinal and experimental designs to further validate these findings and to explore the nuances of how individual power and supportive power interact with physical activity to influence negative emotion. Such research will be pivotal in refining our understanding and in developing targeted interventions to promote mental health and well-being among university students.

## Figures and Tables

**Table 1:** Descriptive analysis and correlation coefficients of demographic and main variables.

Variable	M (SD)	Age	Sex	PA	IP	SP	NE
1. Age	20.32 (1.47)	――					
2. Sex	0.53 (0.66)	0.075	――				
3. PA	16.08 (14.45)	0.003	−0.102	――			
4. IP	51.52 (7.95)	0.046	0.025	0.387 ***	――		
5. SP	41.12 (7.22)	0.028	0.019	0.300 ***	0.569 ***	――	
6. NE	31.11 (26.25)	−0.094	0.073	−0.394 ***	−0.423 ***	−0.464 ***	――

Notes: PA = physical activity; IP = individual power; SP = supportive power; NE = negative emotion; M = mean; SD = standard deviation; *** *p* < 0.001.

**Table 2:** The mediating effect of IP between PA and NE.

Outcome	Predictors	*R*	*R* ^2^	*F*	Beta	*t*	Lower CI	Upper CI
Model 1		0.387	0.150	104.545 ***				
IP	PA				0.387	10.225 ***	0.313	0.461
Model 2		0.491	0.241	94.248 ***				
NE	PA				−0.271	−6.971 ***	−0.347	−0.194
	IP				−0.319	−8.211 ***	−0.395	−0.242

Notes: PA = physical activity; IP = individual power; NE = negative emotion; Beta = standardized. coefficients; CI = confidence intervals; *** *p* < 0.001.

**Table 3:** The moderating effect of SP between PA and NE.

Outcome	Predictors	*R*	*R* ^2^	*F*	Beta	*t*	Lower CI	Upper CI
NE		0.544	0.296	82.814				
	PA				−0.312	−8.205 ***	−0.387	−0.237
	SP				−0.391	−10.758 ***	−0.463	−0.319
	PA × SP				0.089	2.752 **	0.025	0.152

Notes: PA = physical activity; SP = supportive power; NE = negative emotion; Beta = standardized coefficients; CI = confidence intervals; ** *p* < 0.01; *** *p* < 0.001.

## Data Availability

The original contributions presented in this study are included in this article/supplementary material, further inquiries can be directed to the corresponding authors.

## Supplementary Materials

The following supporting information can be downloaded at: https://www.mdpi.com/article/10.3390/healthcare13101170/s1.

## Abbreviations

The following abbreviations are used in this manuscript:

PA	Physical Activity
NE	Negative Emotion
IP	Individual Power
SP	Supportive Power
