# Supplementary material for: Physical Activity Influences Negative Emotion Among College Students in China: The Mediating and Moderating Role of Psychological Resilience
Source: Healthcare (Basel). 2025 May 17;13(10):1170. doi: 10.3390/healthcare13101170 (PMC12110846; doi:10.3390/healthcare13101170)
Supplement: Supplementary file 1 [file healthcare-13-01170-s001.zip › Questionnaire Survey.pdf]

Questionnaire on the Relationship Between Physical Exercise and Negative Emotions Among College Students

Dear College Students,

You are cordially invited to participate in a research study entitled "The Relationship Between Physical Exercise and Negative Emotions in College Students". This investigation has received ethical approval from the Research Ethics Committee of South China University of Technology (Approval No. SCUT-SPT-2022-003). The entire survey will take approximately 20 minutes to complete.We respectfully request that you provide truthful and accurate responses based on your actual circumstances regarding:<1>Your daily physical exercise habits and behaviors; <2>Manifestations of negative emotional experiences; <3>General status of mental health and psychological resilience. Please carefully review the instructions for each section and select the options that most appropriately reflect your situation. All responses will remain strictly confidential and be used solely for academic research purposes. Your participation is invaluable to this research endeavor, and thank you for contributing to the advancement of scientific knowledge in this field.

I. Demographic Information

Age: ( ) years

Gender: ① Male ② Female

II. Physical Exercise Profile

Instruction: The following questions pertain to your physical exercise habits over the past month. Please select the option that best corresponds to your situation.

1. What intensity best describes your physical exercise?

① Light exercise (e.g., walking, stretching, recreational activities)

② Low-intensity non-strenuous exercise (e.g., casual volleyball, jogging, tai chi)

③ Moderate-intensity sustained exercise (e.g., cycling, running, table tennis)

④ High-intensity intermittent exercise with heavy breathing and sweating (e.g., badminton, basketball, tennis, soccer)

⑤ High-intensity prolonged exercise with heavy breathing and sweating (e.g., sprinting,

structured aerobics, swimming)

2. What is the typical duration of each session of the above exercise?

① Less than 10 minutes

② 11-20 minutes

③ 21-30 minutes

④ 31-59 minutes

⑤ 60 minutes or longer

3. How frequently do you engage in the above exercise?

① Once per month

② 2-3 times per month

③ 1-2 times per week

④ 3-5 times per week

⑤ Approximately once daily

III. Assessment of Negative Emotional States (21 Items)

Instruction: Please read each statement carefully and indicate how frequently it applied to you over the past week by selecting the appropriate option. (Please record the numerical value corresponding to your selected option in the parentheses provided after each item.)

0 = Did not apply to me at all

1 = Applied to me to some degree, or some of the time

2 = Applied to me to a considerable degree, or a good part of the time

3 = Applied to me very much, or most of the time

1. **I found it hard to calm myself down.**
2. **I experienced dryness of mouth.**
3. **I could not feel any positive emotions.**
4. **I had difficulty breathing (e.g., breathlessness without physical exertion).**
5. **I found it challenging to initiate tasks.**
6. **I tended to overreact to situations.**
7. **I felt tremors (e.g., hand shaking).**
8. **I felt mentally exhausted.**
9. **I worried about panic-inducing or embarrassing situations.**

10. I felt hopeless about the future.

11. I experienced restlessness.

12. I had difficulty relaxing.

13. I felt depressed and gloomy.

14. I became impatient with interruptions.

15. I felt on the verge of panic.

16. I lost interest in all activities.

17. I felt unworthy as a person.

18. I was easily agitated.

19. I noticed abnormal heartbeats without physical exertion.

20. I felt fearful without apparent reason.

21. I perceived life as meaningless.

IV. Assessment of Psychological Resilience (27 Items)

Instruction: The following statements describe potential attitudes and behaviors when facing adversity. Please select the response that most accurately reflects your experiences.Using a 5-point Likert scale where:

1 = Strongly Disagree 2 = Disagree 3 = Neutral 4 = Agree 5 = Strongly Agree

1. Failures consistently discourage me.

① Strongly Disagree ② Disagree ③ Neutral ④ Agree ⑤ Strongly Agree

2. I struggle to regulate unpleasant emotions.

① Strongly Disagree ② Disagree ③ Neutral ④ Agree ⑤ Strongly Agree

3. I maintain clear life objectives.

① Strongly Disagree ② Disagree ③ Neutral ④ Agree ⑤ Strongly Agree

4. Adversity generally enhances my maturity and competence.

① Strongly Disagree ② Disagree ③ Neutral ④ Agree ⑤ Strongly Agree

5. Setbacks lead me to question my capabilities.

① Strongly Disagree ② Disagree ③ Neutral ④ Agree ⑤ Strongly Agree

6. I lack appropriate confidants during distress.

① Strongly Disagree ② Disagree ③ Neutral ④ Agree ⑤ Strongly Agree

7. I have peer confidants for sharing difficulties.

① Strongly Disagree ② Disagree ③ Neutral ④ Agree ⑤ Strongly Agree

8. My parents respect my perspectives.

① Strongly Disagree ② Disagree ③ Neutral ④ Agree ⑤ Strongly Agree

9. I experience uncertainty in seeking help during crises.

① Strongly Disagree ② Disagree ③ Neutral ④ Agree ⑤ Strongly Agree

10. Growth emerges more from process than outcomes.

① Strongly Disagree ② Disagree ③ Neutral ④ Agree ⑤ Strongly Agree

11. I systematically develop action plans for challenges.

① Strongly Disagree ② Disagree ③ Neutral ④ Agree ⑤ Strongly Agree

12. I internalize emotions rather than verbalize them.

① Strongly Disagree ② Disagree ③ Neutral ④ Agree ⑤ Strongly Agree

13. Adversity possesses motivational value.

① Strongly Disagree ② Disagree ③ Neutral ④ Agree ⑤ Strongly Agree

14. Hardships can facilitate personal development.

① Strongly Disagree ② Disagree ③ Neutral ④ Agree ⑤ Strongly Agree

15. Parental interference dominates my autonomy.

① Strongly Disagree ② Disagree ③ Neutral ④ Agree ⑤ Strongly Agree

16. My voice receives minimal familial acknowledgment.

① Strongly Disagree ② Disagree ③ Neutral ④ Agree ⑤ Strongly Agree

17. Parental support lacks emotional sustenance.

① Strongly Disagree ② Disagree ③ Neutral ④ Agree ⑤ Strongly Agree

18. I proactively seek social support during crises.

① Strongly Disagree ② Disagree ③ Neutral ④ Agree ⑤ Strongly Agree

19. My parents never criticize me harshly.

① Strongly Disagree ② Disagree ③ Neutral ④ Agree ⑤ Strongly Agree

20. I channel full concentration toward problem-solving.

① Strongly Disagree ② Disagree ③ Neutral ④ Agree ⑤ Strongly Agree

21. Negative experiences exhibit psychological persistence.

① Strongly Disagree ② Disagree ③ Neutral ④ Agree ⑤ Strongly Agree

22. Parental encouragement fuels my endeavors.

① Strongly Disagree ② Disagree ③ Neutral ④ Agree ⑤ Strongly Agree

23. I demonstrate efficient emotional recalibration.

① Strongly Disagree ② Disagree ③ Neutral ④ Agree ⑤ Strongly Agree

24. Goal-setting propels my developmental trajectory.

① Strongly Disagree ② Disagree ③ Neutral ④ Agree ⑤ Strongly Agree

25. I perceive latent positivity in all circumstances.

① Strongly Disagree ② Disagree ③ Neutral ④ Agree ⑤ Strongly Agree

26. **Emotional distress triggers communicative avoidance**.

① Strongly Disagree ② Disagree ③ Neutral ④ Agree ⑤ Strongly Agree

27. I experience significant affective instability.

① Strongly Disagree ② Disagree ③ Neutral ④ Agree ⑤ Strongly Agree
