# Supplementary material for: Physical Activity Influences Negative Emotion Among College Students in China: The Mediating and Moderating Role of Psychological Resilience
Source: Healthcare (Basel). 2025 May 17;13(10):1170. doi: 10.3390/healthcare13101170 (PMC12110846; doi:10.3390/healthcare13101170)
Supplement: Supplementary file 1 [file healthcare-13-01170-s001.zip › 调查问卷.pdf]

大学生体育锻炼与负性情绪关系问卷

亲爱的同学：你好！

现邀请你参加一项有关**"大学生体育锻炼与负性情绪关系"**的调查研究。本研究已经得到华南理工大学研究伦理道德委员会的审查许可(批准编号：SCUT-SPT-2022-003)，整个调查时长大约 20 分钟。本次调查需要您根据自身的实际情况，认真并如实回答个人在日常体育锻炼活动情况、负性情绪方面的实际表现，以及有关个人心理健康与心理韧性方面的大概情况。请仔细阅读相关指导语并选出最符合自己的选项。谢谢！

一、人口学情况

1.年龄：（）周岁

2.性别：①男　②女

二、体育锻炼基本情况

指导语：以下是关于最近一个月内的体育锻炼情况，请选择最符合自己的选项。

1.您进行体育锻炼的强度如何?
①轻微运动(如散步、做广播操、打门球等)
②小强度的不太紧张的运动(如消遣娱乐性的打打排球、慢跑、打太极拳等)
③中等强度的较激烈持久运动(如骑自行车、跑步、打乒乓球等)
④呼吸急促，出汗很多的大强度的，但并不持久的运动(如打羽毛球、篮球、网球、足球等)
⑤呼吸急促，出汗很多的大强度的持久的运动(如赛跑、成套健美操练习、游泳等)

2.您在进行上述强度体育活动时，一次多少分钟?
①10 分钟以下
②11 至 20 分钟
③21 至 30 分钟
④31 至 59 分钟
⑤60 分钟以上

3.您进行几次上述体育活动的频率如何?
①一个月 1 次

②一个月 2 至 3 次

③每周 1 至 2 次

④每周 3 至 5 次

⑤大约每天 1 次

三、负性情绪情况（共 21 个题项）

指导语：仔细阅读下面每个句子，“过去一周”您是否出现过这些情况，请选择适合您的选项。(请把选择出的对应分数写在题后。)

0 不符合----1 有时符合---2 常常符合---3 总是符合

1. 我觉得很难让自己安静下来。
2. 我感到口干舌燥。
3. 我好像不再有任何愉快、舒畅的感觉。
4. 我感到呼吸困难（例如：不做运动时也感到气促或透不过气来）。
5. 我感到很难主动去开始工作。
6. 我对事情往往做出过敏反应。
7. 我感到颤抖（例如手震）。
8. 我觉得自己消耗很多精神。
9. 我担心一些令自己恐慌或出丑的场合。
10. 我觉得自己对将来没有什么可盼望。
11. 我感到忐忑不安。
12. 我感到很难放松自己。
13. 我感到忧郁沮丧
14. 我无法容忍任何阻碍我继续工作的事情。
15. 我感到快要恐慌了。
16. 我对任何事也不热衷。
17. 我觉得自己不怎么配做人。
18. 我发觉自己很容易被触怒。
19. 我察觉自己在没有明显的体力劳动时，也感到心律不正常。
20. 我无缘无故地感到害怕。
21. 我感到生命毫无意义。

四、心理韧性情况（共 27 个题项）

指导语：该量表采用 5 点量表级分方法，其标准为“完全不符合”、“比较不符合”、“说不清”、“比较符合”、“完全符合”。以下列出的是当你在生活中经受到挫折打击，或遇到困难时可能采取的态度和做法。请你仔细阅读每一项，然后选择最适合你的答案。

1.失败总是让我感到气馁。

①完全不符合

②比较不符合

③说不清
④比较符合
⑤完全符合

2. 我很难控制自己的不愉快情绪。
①完全不符合
②比较不符合
③说不清
④比较符合
⑤完全符合

3. 我的生活有明确的目标。
①完全不符合
②比较不符合
③说不清
④比较符合
⑤完全符合

4. 经历挫折后我一般会更加成熟有经验。
①完全不符合
②比较不符合
③说不清
④比较符合
⑤完全符合

5. 失败和挫折会让我怀疑自己的能力。
①完全不符合
②比较不符合
③说不清
④比较符合
⑤完全符合

6. 当我遇到不愉快的事情时,总找不到合适的倾诉对象。
①完全不符合
②比较不符合
③说不清
④比较符合

⑤完全符合

7. 我有一个同龄朋友,可以把我的困难将给他/她听。

①完全不符合

②比较不符合

③说不清

④比较符合

⑤完全符合

8. 父母很尊重我的意见。

①完全不符合

②比较不符合

③说不清

④比较符合

⑤完全符合

9. 当我遇到困难需要帮助时，我不知道该去找谁

①完全不符合

②比较不符合

③说不清

④比较符合

⑤完全符合

10. 我觉得与结果相比,事情的过程更能够帮助人成长。

①完全不符合

②比较不符合

③说不清

④比较符合

⑤完全符合

11. 面临困难,我一般会定一个计划和解决方案。

①完全不符合

②比较不符合

③说不清

④比较符合

⑤完全符合

12. 我习惯把事情憋在心里而不是向人倾诉。

①完全不符合

②比较不符合

③说不清

④比较符合

⑤完全符合

13. 我认为逆境对人有激励作用。

①完全不符合

②比较不符合

③说不清

④比较符合

⑤完全符合

14. 逆境有时候是对成长的一种帮助。

①完全不符合

②比较不符合

③说不清

④比较符合

⑤完全符合

15. 父母总是喜欢干涉我的想法

①完全不符合

②比较不符合

③说不清

④比较符合

⑤完全符合

16. 在家里，我说什么总是没人听，

①完全不符合

②比较不符合

③说不清

④比较符合

⑤完全符合

17. 父母对我缺乏信心和精神上的支持。

①完全不符合

②比较不符合

③说不清

④比较符合

⑤完全符合

18. 我有困难的时候会主动找别人倾诉

①完全不符合

②比较不符合

③说不清

④比较符合

⑤完全符合

19. 父母从来不苛责我。

①完全不符合

②比较不符合

③说不清

④比较符合

⑤完全符合

20. 面对困难时，我会集中自己的全部精力。

①完全不符合

②比较不符合

③说不清

④比较符合

⑤完全符合

21. 我一般要过很久才能忘记不愉快的事情

①完全不符合

②比较不符合

③说不清

④比较符合

⑤完全符合

22. 父母总是鼓励我全力以赴

①完全不符合

②比较不符合

③说不清

④比较符合

⑤完全符合

23. 我能够很好的在短时间内调整情绪

①完全不符合

②比较不符合

③说不清

④比较符合

⑤完全符合

24. 我会为自己设定目标，以推动自己前进。

①完全不符合

②比较不符合

③说不清

④比较符合

⑤完全符合

25. 我觉得任何事情都有积极的一面。

①完全不符合

②比较不符合

③说不清

④比较符合

⑤完全符合

26. 心情不好也不愿意跟别人说。

①完全不符合

②比较不符合

③说不清

④比较符合

⑤完全符合

27. 我情绪波动很大，容易大起大落

①完全不符合

②比较不符合

③说不清

④比较符合

⑤完全符合
